# Conventional surgical repair of traumatic rupture of the thoracic aorta

**DOI:** 10.1007/s11748-014-0422-x

**Published:** 2014-12-01

**Authors:** Kiyoshi Chiba, Hiroyuki Abe, Yosuke Kitanaka, Takeshi Miyairi, Haruo Makuuchi

**Affiliations:** 1 Department of Cardiovascular Surgery, St. Marianna University School of Medicine, 2-16-1 Sugao Miyamaeku, Kawasaki, Kanagawa 216-8511 Japan; 2 Department of Cardiovascular Surgery, St. Marianna University School of Medicine, Yokohama Seibu Hospital, Yokohama, Kanagawa Japan

**Keywords:** Traumatic aortic rupture, Revised Trauma Score (RTS), Injury Severity Score (ISS), Surgical repair

## Abstract

**Objective:**

Traumatic rupture of the thoracic aorta is a life-threatening injury requiring urgent surgical intervention. Despite recent improvements in resuscitation and emergency operative techniques, the outcomes of patients with multiple injuries are still associated with a high mortality rate. We retrospectively examined the preoperative demographic data, associated complications and mortality rate of these patients.

**Materials and methods:**

We analyzed the data (1991–2009) of 18 patients with acute traumatic rupture of the thoracic aorta. Most patients had rupture limited to the aortic isthmus and severe associated injuries in other organs. The aorta was repaired by direct suturing, patch plasty (*n* = 5; 27.7 %) or graft interposition (*n* = 9; 50 %).

**Results:**

The overall mortality rate was 33.3 %. All six patients who underwent emergency surgery within 2 h died, four intra-operatively and two postoperatively. The causes of the intra-operative mortality were uncontrollable hemorrhage and irreversible cardiac arrest due to penetrating injury of the thoracic aorta and intercostal arteries in three patients, and uncontrollable hemorrhage due to severe liver laceration in one. The surgical complications (42.8 %) were acute lung injury (*n* = 2), liver insufficiency (*n* = 2), acute renal failure (*n* = 1) and cerebral infarction (*n* = 1). No patients had postsurgical paraplegia. The mean period between arrival and treatment and the mean Injury Severity Score were significantly higher in group D than in group A.

**Conclusion:**

To improve the outcome of traumatic thoracic aortic injury, the degree of multi-organ damage, the priority of treatment be evaluated accurately is important.

## Introduction

Traumatic rupture of the thoracic aorta is severe injury with high mortality.

In the recent reports, more than 80 % of the patients with severe injuries die from lethal exsanguinations at the scene of the accident; only 15–20 % are transported to the hospital alive [1, 2]. Statistical data on trauma in Japan are few, but more than 40,000 people are reported to die every year due to trauma in Japan [3].

According to the Japan trauma databank, the most frequent cause of death is a blunt trauma by traffic accident (39.2 %) or fall (19.9 %). The sites of trauma are the head in 21.1 %, the chest in 15.3 % and the lower extremities in 21.1 %. Hence, traumatic rupture of the thoracic aorta in Japan is caused mainly by blunt trauma.

Prompt surgical repair for thoracic aortic injury is generally recommended. If the patient has more immediate life-threatening injuries that require emergency laparotomy or craniotomy, however, the aortic repair may be delayed according to the guidelines 2000 Eastern Association for Surgery of Trauma (EAST) [4].

In patients with multiple injuries, it is reported that better results are obtained by treating severe and more urgent extra-aortic trauma to stabilize the general condition before performing the aortic repair [4].

On the other hand, despite developments in trauma management and operative techniques, still remains high mortality.

The purpose of this retrospective study was to consider the associations of the preoperative demographic data, such as Injury Severity Score (ISS) [5], and physiologic scoring system (Revised Trauma Score, RTS) [6] in acute settings, to determine surgical strategies to reduce the incidence of mortality. ISS, which proposed by Baker et al. in 1974, has become world-wide standard for to determine the severity of injury. Calculation of the ISS is severity scores based on contains the Abbreviated Injury Scale (AIS) [5].

## Patients and methods

We analyzed the data of 18 patients with acute traumatic rupture of the thoracic aorta who had undergone treatment at our institution between September 1991 and November 2009. Data were obtained by retrospectively reviewing medical records and operative reports. The age range was from 17 to 81 years (mean, 43.8 ± years). All patients had blunt chest trauma involving sudden deceleration (traffic accidents, 72.2 %) or a fall from a great height (27.8 %).

A diagnosis of traumatic rupture of the thoracic aorta was established by chest X-ray imaging, together with computed tomography (CT) or angiography [7–9]. The diagnosis by CT required clear signs of aortic injury such as pseudoaneurysm, intimal flap, aortic contour abnormality, intraluminal thrombus or pseudocoarctation.

Most of the patients had a typical rupture of the aortic isthmus (76.5 %), and 5 had a rupture of the descending aorta (23.5 %). Table 1 shows the demographic and clinical data of the patients. All patients had associated injuries in various organs; there were additional severe lesions, including craniocerebral injury in 6, lung contusion with reduced respiratory function in 1 and hemopneumothorax in 12, injury to multiple extremities in 9, and pelvic fractures in 6, as visceral organ injuries in the trunk, liver laceration in 4, renal laceration in 3, spleen laceration in 1, and injury to superior mesenteric artery and small intestine in 1 (Table 2).

**Table 1:** Pre- and postoperative clinical data of patients

No	Sex	Age	RTS	ISS	Body area of AIS > 3	Procedure	Outcome
1	F	56	5.24	50	Head (3), chest (5), extremity (4)	Direct closure → TAE	Alive
2	M	25	5.24	35	Chest (5), extremity (3)	Grafting	Alive
3	M	47	5.97	50	Chest (5), abdominal (3), extremity (4)	TAE → direct closure	Alive
4	M	63	7.11	35	Chest (5), extremity (3)	Direct closure	Alive
5	M	25	4.94	50	Head (3), chest (5) extremity (4)	TAE → direct closure	Alive
6	M	22	3.57	43	Head (3), chest (5) abdominal (3)	Grafting + small bowl resection	Alive
7	M	39	7.84	26	Chest (5)	Grafting	Alive
8	M	45	3.07	50	Head (3), chest (5) extremity (4)	TAE → Grafting pelvic external fixation	Alive
9	M	18	7.84	43	Chest (5), abdominal (3), extremity (3)	Grafting	Alive
10	M	38	7.11	43	Head (3), chest (5), extremity (3)	Grafting + femoral fixation	Alive
11	M	18	7.84	35	Chest (5), extremity (3)	Grafting + femoral fixation	Alive
12	M	19	7.84	30	Chest (5)	Grafting	Alive
13	F	69	4.5	65	Chest (5), abdominal (6)	Partial hepatectomy ET (direct closure)	Dead
14	M	17	7.55	45	Chest (5 → 6), extremity (3)	ET	Dead
15	M	20	4.5	75	Chest (5), abdominal (5), extremity (5)	TAE → grafting	Dead
16	M	81	5.5	70	Head (3), chest (5), abdominal (6)	EL + ET	Dead
17	M	17	5.3	45	Head (3), chest (5 → 6)	ET	Dead
18	M	18	6.9	45	Head (3), chest (6)	ET	Dead

Injury Severity Score is allocated to each of the six body regions [head, face, chest, abdomen, extremities (including the pelvis) and external]. Only the highest Abbreviated Injury Score (AIS) in each body region is used as a final score (aortic injury is excluded in the calculation of chest AIS). The scores of the three most severely injured body regions are squared and summed to produce the final ISS

*TAE* transcathetral arterial embolization, EL emergency laparotomy, *ET* emergency thoracotomy, *RTS* Revised Trauma Score, *ISS* Injured Severity Score

**Table 2:** Associated injuries (body area of AIS > 3)

N	Head and neck	Face and chest	Abdomen	Extremity (including pelvis) external
1	Subdural hematoma	Rib fractures		Femoral fractures
2		Hemopneumothorax		Femoral fractures
3		Tension pneumothorax	Spleen lacerations Kidney lacerations	Pelvic fractures
4		Rib fractures		Femoral fracture
5	Cerebral contusion	Hemopneumothorax lung contusion		Pelvic fractur
6	Cerebral contusion Cervical fracture	Rib fractures	Injury to superior mesenteric artery Injury to small intestine	Extremity fracture
7	Facial laceration	Rib fractures		
8		Rib fractures		Pelvic fracture Extremity fracture
9		Hemopneumothorax	Kidney laceration	Femoral fracture
10	Cerebral contusion	Hemopneumothorax	Liver laceration	Femoral fracture
11	Facial laceration	Hemopneumothorax		Pelvic fracture
12		Hemopneumothorax		Extremity fracture
13		Sever pulmonary insufficiency	Liver ulceration	
14		Hemopneumothorax		Femoral fracture
15		Hemopneumothorax Rib fractures	Liver lacerations Kidney ulceration	Pelvic fracture
16		Hemopneumothorax	Liver laceration	Pelvic fracture
17	Cerebral contusion	Hemopneumothorax		
18	Vertebral fracture Spinal injury	Hemopneumothorax Rib fractures		

For the anatomic scoring system, we used the ISS, with a range from 0 to 75. ISS is considered to correlate with mortality from external wounds. The ISS is allocated to each of the 6 body regions: head, face, chest, abdomen, extremities (including the pelvis) and external regions. The highest Abbreviated Injury Score (AIS-90) of each body region is used (Table 3). The scores of the 3 most severely injured body regions are squared and added together to produce a final ISS (Table 1) [5].

$$< {\text{ISS}} = \left( {\text{highest AIS}} \right)^{2} + \left( {2{\text{nd}}\;{\text{AIS}}} \right)^{2} + \left( {3{\text{rd}}\;{\text{AIS}}} \right)^{2} >$$

**Table 3:** Anatomic scoring system

Abbreviated Injury Score (AIS)-90
AIS 1 = minor
AIS 2 = moderate
AIS 3 = serious (nonlife-threatening injury)
AIS 4 = severe (life-threatening but survival)
AIS 5 = critical, survival uncertain
AIS 6 = maximum (currently untreatable)

AIS is allocated to each of the six types of injury

The physiologic scoring system (RTS) is used to determine the rate of survival according to consciousness, systolic blood pressure and respiratory rate scores (Table 4) [6].

**Table 4:** Physiologic scoring system

Revised Trauma Score (RTS)			
GCS	SBP	RR	Score
13–15	≥90	10–29	4
9–12	76–89	≥30	3
6–8	50–75	6–9	2
4–5	1–49	1–5	1
3	0	0	0

Revised Trauma Score (RTS) is allocated to each of the five points on the Glasgow coma scale, systolic blood pressure and RR (respiration rate). These three scores are then summed to produce the RTS

RTS = 0.9368 × GCS Score + 0.7326 × SBP Score + 0.2908 × RR Score

*GCS* Glasgow Coma Scale, *SBP* systolic blood pressure, *RR* respiratory rate

### Treatment

We aimed to perform surgical repair immediately after admission, unless severe associated injuries or complications restricted treatment options. Hypertension was prevented by deep sedation and treated if necessary with the calcium-blocking drug and/or nitroprusside.

In the present study, 18 patients underwent surgical repair for traumatic aortic rupture. Of these patients, 17 (94.4 %) were treated within 7 days; in nine patients, emergency surgery was performed within 6 h after the trauma. Four patients underwent surgery without cardiopulmonary bypass Femoro–femoral bypass was established with systemic heparinization in four patients. A minimal amount of heparin was administered to maintain cardiopulmonary bypass keeping activated coagulation time (ACT) at approximately 200–250 s. In ten patients, left heart bypass with the Bio-Pump (Bio-Medicus, Minneapolis, MN, USA) was performed with argatroban, an antithrombin agent. Four patients with critical intra-abdominal injuries or pelvic fractures on arrival had to undergo immediate transcatheter arterial embolization (TAE) of the bleeding arteries before surgical repair. In one of these patients, surgery was delayed for more than 20 days after the initial trauma due to severe pulmonary contusion and severe inflammation after TAE of the bilateral internal iliac arteries. In another patient with pelvic fracture, the bleeding aggravated after surgical repair of traumatic aortic rupture, and therefore, we had to immediately perform TAE of the internal iliac arteries.

In all cases, the thoracic aorta was approached via a lateral or posterolateral thoracotomy, with an incision in the fourth left intercostal space. The aorta was dissected and isolated circumferentially proximal and distal to the injured segment and then clamped proximally between the left common carotid artery and the left subclavian artery. In five patients (27.7 %), the aorta was repaired by direct suturing or patch plasty, and in nine patients (50 %), a tube-graft was interposed. After weaning from bypass, the effects of heparin were reversed with protamine sulfate. The remaining four patients (22.3 %) underwent emergency thoracotomy and laparotomy in outpatient room due to the hemorrhagic shock of bleeding and passed away before aortic reconstruction.

Postoperatively, patients were transferred to the intensive care unit with continuous monitoring of arterial and central venous pressure, renal function, consciousness and other hemodynamic and clinical parameters. Follow-up was conducted by office visits and hospital reports.

A *p* value of <0.05 was considered to represent a statistically significant difference on analysis with the Student’s *t* test and Welch test (Table 5).

**Table 5:** Patient outcomes

	Alive (*n* = 12)	Dead (*n* = 6)	*p*
Age (years [mean]/range)	34.6 ± 15.7 (18–63)	37 ± 29.7 (17–81)	0.86
Period between trauma and arrival	411 ± 1,179.5 min (28 min–3 days)	70.5 ± 83.6 min (9–255 min)	0.81
Period between arrival and treatment	47.7 ± 130.4 h (1.8 h–20 days)	2.6 ± 1.8 h (0.5–5.3 h)	0.02
ISS [mean]/(range)	40.8 ± 8.5 (26–50)	57.5 ± 14.1 (34–75)	0.006
RTS [mean]/(range)	6.13 ± 1.72 (3.07–7.84)	5.7 ± 1.26 (4.5–7.55)	0.59

## Results

We divided all patients into either group A (alive; *n* = 12) or group D (dead; *n* = 6) (Table 5). We then estimated possible correlations among age, period between wounding and arrival at hospital, period between arrival at the hospital and operation, and preoperative clinical data. The average patient age was 34.6 ± 15.7 years (range 18–63 years) in group A and 37 ± 29.7 years (range 17–81 years) in group D (*p* = 0.86). The mean period ± SD between injury and arrival at the hospital was 413.5 ± 1,179.5 min (median 32.5 min; range 28 min–3 days) in group A and 70.5 ± 83.6 min (median 41.5 min; range 9–255 min) in group D (*p* = 0.81). The mean period between arrival at the hospital and operation was 47.9 ± 130.4 h (median 9.1 h; range 1.8 h–20 days) in group A and 2.6 ± 1.8 h (median 1.9 h; range 0.5–5.3 h) in group D (*p* < 0.02). The RTS was 6.13 ± 1.72 in group A and 5.7 ± 1.26 in group D (*p* = 0.59). The ISS (AIS > 3) was 40.8 ± 8.5 in group A and 57.5 ± 14.1 in group D (*p* = 0.006). There were no statistically significant differences in the data between the two groups, except for the mean period between arrival and treatment, and the mean ISS. Particularly, the mean ISS was significantly higher in group D than in group A. Moreover, the multivariate analysis was further conducted for these results, and ISS showed accuracy as a prognosis predictive factor with the ROC curve (Table 6).

**Table 6:** Evaluation of ISS by multivariate analysis and ROC curve

	*p* value	Odds	95 % CI
ISS	0.0001	2.24	1.22–20.06
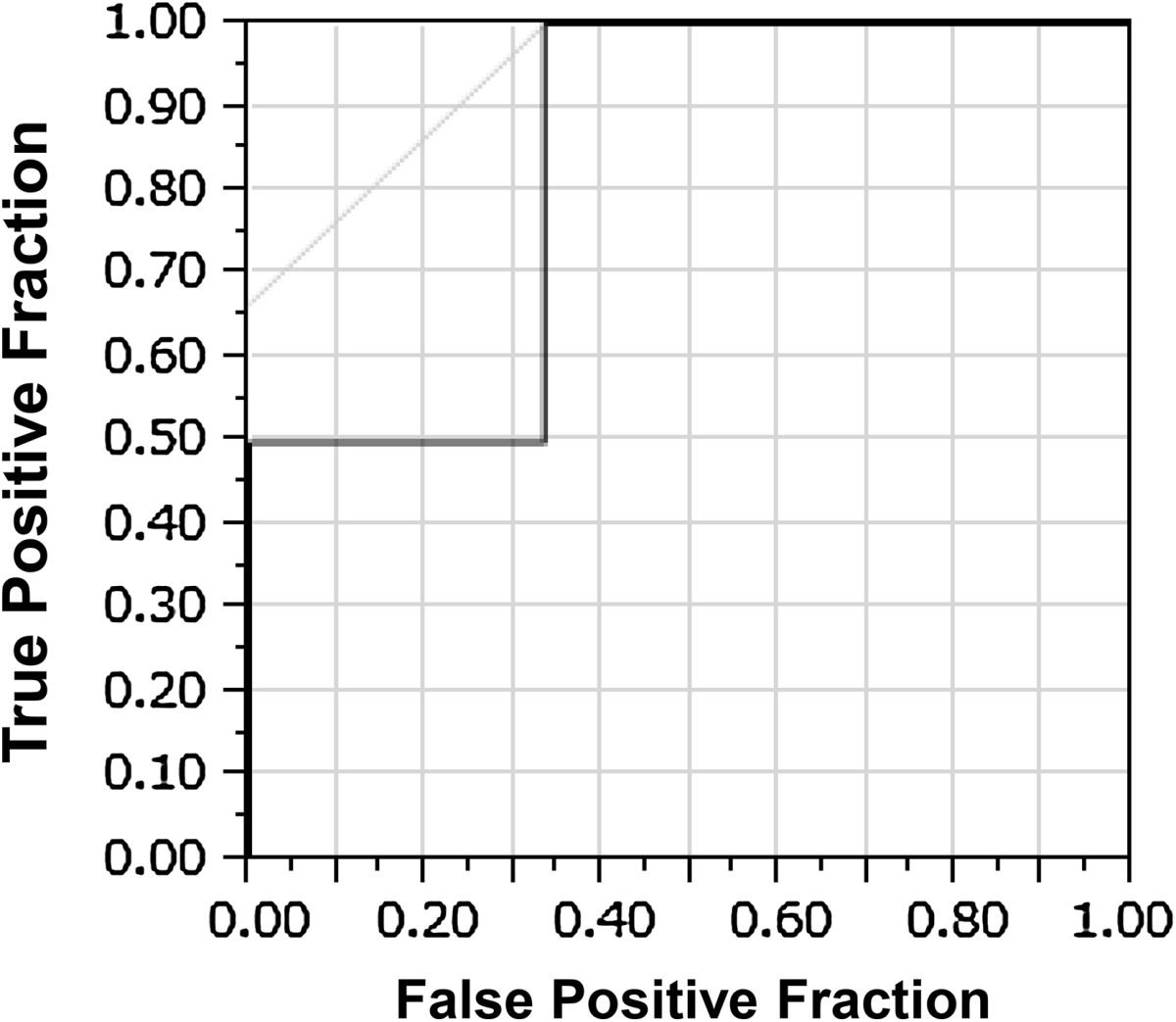			

AUC = 0.833

ISS cut off point = 45.0

*CI* confidence interval

The overall mortality rate was 33.3 %. All six patients who underwent emergency surgery within 2 h died, four intra-operatively and two postoperatively. The causes of the intra-operative mortality were uncontrollable hemorrhage and irreversible cardiac arrest due to penetrating injury of the thoracic aorta and the intercostal arteries in three patients, and uncontrollable hemorrhage due to severe liver laceration in one.

The rate of postoperative mortality was 14.3 % (2/14). One patient died of hemorrhagic shock due to disseminated intravascular coagulation and critical liver damage on day 2, and the other patient died of severe pulmonary insufficiency and liver damage on the day of operation.

Eleven patients had complicated blunt torso injuries. Their causes were traffic accidents (*n* = 6) and a fall from a great height (*n* = 5).

In six patients (42.8 %), serious complications developed after surgical repair, namely acute lung injury (*n* = 2), liver insufficiency (including myonephropathic metabolic syndrome) (*n* = 2), acute renal failure (*n* = 1) and cerebral infarction (*n* = 1). Paraplegia associated with surgery did not occur in any patient.

The average hospital stay was 101.8 ± 47.6 days (median 81 days, range 16–263 days). Among the patients in group A, four with brain injuries were transferred to other hospitals for rehabilitation. The remaining eight patients were doing well at the time of writing.

## Discussion

We examined the preoperative demographic data, associated complications and mortality rate of patients with traumatic rupture of the thoracic aorta in acute settings. Acute rupture of the thoracic aorta due to blunt chest trauma is extremely lethal. Death occurs in up to 85 % of the cases before arrival at a hospital [10–12].

Its etiology, patient status at the time of treatment and associated injuries are mainly responsible for the prognosis. Despite recent advances in surgical techniques and management, including one-lung ventilation and the use of cardiopulmonary bypass, surgical repair of the aorta is associated with high mortality. There is a general consensus concerning the necessity of surgery in cases of acute aortic rupture, but the ideal timing of repair is still controversial. In particular, the coexistence of lung contusion, intracranial, intra-abdominal or pelvic injuries and possible aggravation of extrathoracic bleeding by heparinization during aortic repair make it difficult. Therefore, delayed repair of traumatic aortic rupture is a surgical option for critically traumatic patients at risk of nonaortic associated injuries [13, 14]. In our experience, we suggest performing surgery of the aortic rupture after definite diagnosis of other actively bleeding injuries, especially in intra-abdominal organs. On the other hand, we experienced that re-rupture can result in death even during a short waiting period and under controlled blood pressure. It is therefore difficult to determine the priority of treatment of multiple injuries.

Considering the results of this study, the period between arrival at a hospital and the beginning of surgery may not be directly associated with the result. More favorable results were suggested to be obtained in cases where enough time was allowed to consider various treatment options.

To date, three different surgical techniques have been used for the repair of aortic rupture: the simple clamp and sew procedure, repair with distal perfusion via left heart bypass and femoro–femoral partial cardiopulmonary bypass. The effects of systemic heparinization on mortality and the risk of hemorrhage in such patients with multiple injuries, particularly in those with coexisting brain or pulmonary contusions, remain unclear [15–17], because no separate analysis has been made of the different forms of distal perfusion. The standard assisted circulation technique in our institution is left heart bypass using a Bio-Pump (Bio-Medicus) with argatroban, an antithrombin agent [18, 19]. However, we used femoro–femoral cardiopulmonary bypass with low-dose heparin for those cases in which clamping the proximal site was difficult due to dissection or external hematoma, or for those with unstable respiration due to severe lung contusion.

We found that ISS was significantly higher in group D than in group A. As the rate of complications were higher in patients with ISS higher than 50, estimating ISS may be useful in predicting the outcome (Table 6).

Neurological complications remain a major challenge after the surgical repair of the thoracic aortic rupture. In particular, open surgery of the thoracic aorta with craniocerebral injuries comprises about 9–19 % of neurological events [20, 22]. In the current series, we experienced one patient with combined left hemiparalysis, but none with postoperative paraplegia and paraparesis. According to recent reports, the use of endovascular stentgrafts for traumatic aortic rupture reduces the risk of paraplegia and neurological events [23–25]. Endovascular stent-grafting has several benefits for polytrauma patients, such as rendering cardiopulmonary bypass unnecessary. Moreover, nonperformance of thoracotomy is associated with less bleeding, as well as shorter time for operative treatment for associated injury, which results in better clinical outcomes [24]. In 2004, Forbes et al. [25] reported that on performing laparotomy, continuous intra-abdominal bleeding was controlled by splenectomy and that they exposed the infrarenal aorta as an access route for endovascular repair of thoracic aortic injury. In this context, endovascular treatment for acute traumatic aortic rupture is feasible and can be a valid alternative to conventional open surgery in selected patients. To improve surgical mortality, we think that endovascular repair should be performed first or simultaneously with other surgical procedures if the patient is elderly or has intra-abdominal hemorrhage due to liver laceration (AIS > 3) or other vascular injuries in the torso.

However, the possible problem of endovascular treatment remains considering the risk of serious device-related complications, whether in the short, medium or long term [24, 25]. At any rate, our institution did not adopt endovascular treatment then, and in the present study, we were unable to compare the efficacy of conventional surgical repair and endovascular treatment.

The data are small, and the study is retrospective and nonrandomized. As this study has such limitations, we could not definitively establish the superiority of any one technique over another.

## Conclusion

To improve the outcome of traumatic thoracic aortic injury, the degree of multi-organ damage, the priority of treatment be evaluated accurately is important.

Patients whose ISS was higher due to uncontrollable bleeding because of intra-abdominal critical injury or blunt injuries of the thoracic aorta had poorer outcomes following conventional surgical repair by direct suturing, patch plasty or graft interposition. Therefore, we have introduce endovascular treatment and are examining the efficacy of endovascular repair for the patients whose ISS was high, or who were considered high risk for conventional repair.
